# What Unfavorable Factors Are Associated with Low Serum Total Cholesterol in a Japanese Population?

**DOI:** 10.2188/jea.12.271

**Published:** 2007-11-30

**Authors:** Xu Mao, Tomonori Okamura, Sohel R Choudhury, Yoshikuni Kita, Takashi Kadowaki, Akira Okayama, Isao Niki, Hirotsugu Ueshima

**Affiliations:** 1 Department of Health Science, Shiga University of Medical Science, Shiga, Japan.; 2 Department of Hygiene and Public Health, School of Medicine, Iwate Medical University, Iwate, Japan.; 3 Honorary Director, Shiga National Hospital, Shiga, Japan.

**Keywords:** cholesterol, nutrition survey, drinking, vitamin A, blood transfusion, smoking

## Abstract

An inverse association between blood cholesterol level and excess mortality in low cholesterol level subjects has been reported, but there has been no reasonable explanation widely accepted. To evaluate the associations between unfavorable factors and low blood cholesterol in non-Western populations, we performed a cross-sectional study in a rural Japanese population. A self-administered questionnaire concerning health characteristics and a nutritional survey, using a continuous 48-hour dietary record, was conducted on 461 males and 571 females aged 20-79 years old. The serum total cholesterol (TC) of less than 160 mg/dl was defined as low cholesterol, which accounted for 18% of the subjects. The multivariate odds ratio of having low cholesterol adjusted for age and selected variables were 0.70 (95% CI: 0.52-0.94) for 1SD increment of Key’s lipid factor, 0.71 (0.51-0.97) for 1SD increment of vitamin A intake, 2.23 (1.01-4.91) for heavy drinking, 2.80 (1.21-6.46) for being underweight and 2.59 (1.01-6.61) for blood transfusion in males, and 1.04 (1.00-1.08) for 10 cigarette-year increase in smoking in females. Even when further adjusted for body mass index, these associations were still significant except for those who were underweight and had undergone blood transfusion in males. These findings may partly explain the excess mortality of the Japanese males with low serum TC.

## INTRODUCTION

Low blood cholesterol level has been reported to be associated with an excess number of deaths from a variety of causes in many studies^1^^-^^7^^)^. There are several potential mechanisms for this association, but no explanation has been widely accepted. Several studies on the relation between cholesterol and antioxidant vitamin such as vitamin A have suggested that hypocholesterolemia may be associated with a low serum antioxidant reserve, possibly increasing susceptibility to oxidative stress^8^^,^^9^^)^. An inverse relation with cancer was stronger with serum retinol than with cholesterol, which suggested that the association with cholesterol might be secondary^10^^)^. Furthermore, in Western populations including Japanese-American males, subjects with low serum TC were found to have several adverse health characteristics such as smoking, heavy drinking, low body mass index (BMI), anemia, peptic ulcer, certain gastrointestinal conditions^11^^,^^12^^)^. However, in Asian populations including Japanese, whose lifestyles are different from Western people, there have been few studies on the associations of low serum TC with lifestyle factors including intakes of antioxidant vitamins. More research is needed to clarify these associations. We explored the associations of low serum TC with lifestyle factors including vitamin intake, past history of disease and biological factors in a rural Japanese population.

## MATERIALS AND METHODS

### Study Population

The present study was a cross-sectional survey designed to investigate the characteristics of lifestyle, past history of disease and biological factors associated with low serum TC in Konan Town, Shiga Prefecture, in a rural community of west Japan. We randomly selected one area from Konan Town’s 25 administrative divisions each year, and conducted the clinical examinations and nutrition survey of all residents aged 19 years and over except for employees and students during each spring season between 1987 and 1995 with the exception of 1994. A total of 1,069 residents were surveyed during these eight years. The response rate was 80% for the study population and 37% for the whole population in these study areas. We selected the 1,032 residents aged 20-79 years as the subjects of the present study. All subjects had complete data needed for the analysis.

### Clinical Examinations

Blood pressures were measured by trained public health nurses using standard mercury sphygmomanometers on the right arm of sitting participants after a 5-minute rest. The height and weight of subjects were measured wearing light clothes in their stocking feet. A nurse interviewed each subject regarding whether they had breakfast. Overnight fasting blood of the subjects was drawn and serum TC, high density lipoprotein cholesterol (HDL-C), triglyceride, total protein, glucose and blood hemoglobin were determined in one laboratory (Medic, Shiga). Serum TC was measured by an enzymatic method and validated through a lipid standardization program by Osaka Medical Center for Cancer and Cardiovascular Diseases, which is a member of the Cholesterol Reference Method Laboratory Network (CRMLN) controlled by the Centers for Disease Control and Prevention, Atlanta^13^^)^. Deviations of means were <2% and coefficients of variation were <1%. We used the Friedewald’s formula to calculate the low density lipoprotein cholesterol (LDL-C) of subjects whose serum triglyceride level was <400 mg/dl^14^^)^.

### Nutrition Survey

The nutrition status of each subject was evaluated using the 48-hour dietary record method. The subjects were asked to note everything they ate and drank during the 48 hours in a food record diary. Visual charts describing the portion size and weight of various kinds of food and their ingredients were also given. The subjects were also asked to note the weight of the food items comparing them with the chart. A trained dietician checked the diary for each individual when it was returned and any discrepancy was corrected after discussing it with the subject. The nutrition survey data were calculated using computer software in accordance with the standard Japanese food table (4th edition)^15^^)^. The present survey method was based on the protocol of the National Nutrition Survey in Japan^16^^)^. The average energy (E) and intakes of proteins, fats, carbohydrates, alcohol, saturated fatty acids (S), polyunsaturated fatty acids (P), dietary fiber, dietary cholesterol (C), and vitamins A and C were calculated. The dietary lipid factor of Keys was calculated as [(S-P/2) × 2430/E+1.5(1,000 C/E)^1/2^]^17^^)^.

Alcohol intake was determined from the food record diary. The subjects were asked to record the amount of “sake” (Japanese traditional alcohol beverage), beer, whisky and other alcoholic beverages as the number of “go” (a Japanese traditional unit of volume, 1 “go” =180 ml) of “sake”. Responses were converted to the total amount of “go” units and were converted into pure alcohol equivalents (1“go” = 23 g of ethanol).

### Definition of Study Variables

Since previous findings indicated that serum TC <160 mg/dl was significantly associated with increased risk of all-cause mortality in an MRFIT study^1^^)^ and cancer mortality in the Japanese follow-up study^7^^)^, we selected 160 mg/dl and 200 mg/dl of serum TC as cut-off points and defined serum TC <160 mg/d (<4.14 mmol/l) as low serum TC in the present study. All subjects were divided into three groups with different serum TC levels. There were 184 subjects with serum TC <160 mg/dl (18% of the study population), 478 subjects with 160-199 mg/dl serum TC (46%) and 370 subjects with serum TC ≧200 mg/dl (36%).

A face-to-face interview with a public health nurse was conducted to ascertain the smoking and drinking status. The smoking status was classified as never, past and current smoking. Furthermore, total cigarette-years of smoking were calculated as the numbers of cigarette smoked per day multiplied by years of cigarette use. The drinking status was divided into three categories: never, occasional and daily drinking.

The median alcohol consumption determined from the food record diary in this population was 12.2 g/day in the entire population, 24.6 g/day in males and 2.3 g/day in females. Heavy drinkers were defined as those whose alcohol consumptions were beyond 95 percentile in the whole population and 90 percentile in males. This category included those who reported to be “daily drinkers” of consumption ≧56 g/day (2.5 “go” /day). The BMI was calculated as weight (kg) divided by the square of height (m). Means and standard deviations (SD) of BMI were 22.0 (3.0) kg/m^2^ in both males and females. Underweight was defined as BMI <18.5 kg/m^2^ according to the World Health Organization guidelines^18^^)^. The subjects who were underweight were about 10 percent of the whole population.

The self-reported past history of disease was ascertained through an interview with a medical doctor and a public health nurse. Since it is difficult to distinguish the self-reported present histories of diseases from the self-reported past histories, they were combined as “the past histories of diseases” in the present study. We selected those diseases with greater than 10 subjects (about one percent of the study population) as study variables to analyze the relation between low serum TC and past history of disease. The selected diseases included peptic ulcer, diabetes, liver disease, anemia, kidney disease and hypertension. Since surgery and blood transfusion were associated with a past history of severe disease, they might have affected the cholesterol metabolism of the body; these factors were also selected as study variables, although we did not identify the cause of surgery and blood transfusion. There were 8 subjects with a past history of stroke and 2 subjects with a past history of coronary heart disease in 1,032 subjects. We could not find any subject with a reported past history of cancer in this study population.

### Statistical Analysis

Males and females were analyzed separately, because their mean serum TC were obviously different, and some studies reported a stronger relation between low cholesterol and excess mortality in males than in females^2^^,^^7^^)^.

SPSS version 9.0 software was used for all data analysis^19^^)^. One-way analysis of variance for continuous variables and the chi-square test for categorical variables were conducted for statistical analysis between groups. Further analysis was done with multiple logistic regressions. Low serum TC was determined as a dependent variable (0=no, 1=yes). The health-related factors, which indicated significance or no significance but showed a linear association for trend by univariate analysis, were selected as independent variables. These factors were divided into three groups on the basis of commonality of function, and we performed three multivariate analyses.

In model 1, we analyzed the lifestyle factors including Keys’ lipid factor and intakes of either vitamin A or vitamin C, total cigarette-years of smoking (10 cigarette-years), heavy drinking and underweight (0=no, 1=yes) as independent variables. We did not included vitamin A and vitamin C in one model, because these vitamins had a significant positive correlation (R=0.37, P<0.001). Furthermore, with respect to the intake of vitamins A and vitamin C, we also used the energy-adjusted intake levels of these vitamins as independent variables instead for crude values.

In model 2, a past history of peptic ulcer, kidney disease, anemia, liver disease, diabetes, hypertension, surgery and blood transfusion were included as independent variables (0=no, 1=having).

In model 3, biologic factors of systolic blood pressure, hemoglobin, triglyceride, HDL-C, total protein and glucose were included as independent variables. Each analysis was done in triplicate: adjusted for selected variables; selected variables adding age; selected variables adding age and BMI. But underweight as a covariate in model 1 was not adjusted for BMI. We used a standard deviation of each continuous variable for calculating increments in odds ratios in models 1 and 3. Concerning smoking, we used 10 cigarette-years for calculating increments in the odds ratio. All probability values were two-tailed.

## RESULTS

**Figure 1.**
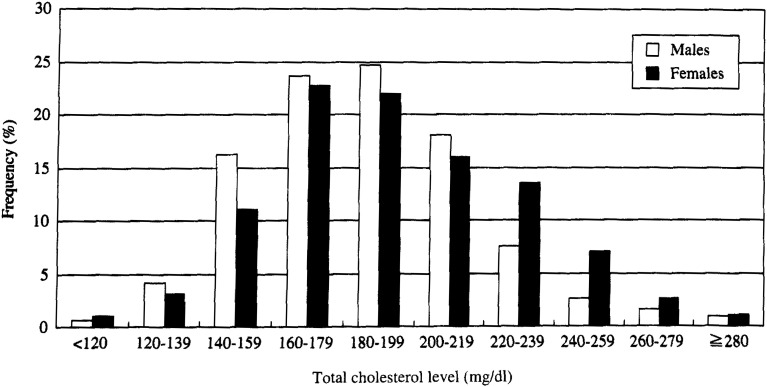
Distribution of total cholesterol levels among 1,032 subjects aged 20-79 years old in Konan Town, Shiga prefecture, Japan, between 1987-1995 with the exception of 1994.

Figure 1 shows the distribution of senim TC categorized into 10 levels of 20 mg/dl intervals in the present study population. The mean serum TC (±standard deviation; SD) were 190 (±33.5) mg/dl in the whole population, 185 (±31.2) mg/dl in males and 194 (±34.8) mg/dl in females.

The gender-specific population characteristics stratified by serum TC level are shown in Table 1. Subjects with low serum TC had the lowest level of Keys’ lipid factor, intakes of cholesterol, vitamin A and vitamin C, and the lowest rate of hypertension in both genders for trend. They also had the highest rate of heavy drinker, underweight, liver disease, anemia and blood transfusion in males, and cigarette smoking, peptic ulcer, anemia and blood transfusion in females. Furthermore, they had the lowest mean levels of systolic and diastolic blood pressure, LDL-C, triglyceride, glucose, total protein and hemoglobin in both genders among the three groups stratified by serum TC.

**Table 1.** Population characteristics by gender and serum total cholesterol levels among 1,032 subjects aged 20-79 years old in Konan Town, Shiga, Japan, between 1987-1995 with the exception of 1994.

	Males				Females			
	
	Serum TC level (mg/dl)			P*	Serum TC level (mg/dl)			P*
	
	<160	160–199	≧200		<160	160–199	≧200	
Number of subjects	97	223	141		87	255	229	
Age (year)	48.1	49.1	49.1	0.571	42.9	47.2	55.9	<0.001
Body mass index (kg/m^2^)	20.8	22.0	23.0	<0.001	20.9	21.8	22.6	<0.001
Lifestyle factors								
Intake of major nutrients								
Energy (kcal/day)	2,376	2,313	2,313	0.346	1,797	1,799	1,784	0.790
Protein (g/day)	84.6	85.8	87.5	0.317	67.6	67.9	68.1	0.823
Fat (g/day)	55.1	55.4	57.6	0.282	47.6	47.9	46.3	0.492
Carbohydrate (g/day)	337	324	322	0.149	270	269	271	0.895
Alcohol (g/day)	27.5	24.8	22.3	0.185	2.6	2.7	1.6	0.208
Saturated fatty acid (g/day)	13.9	14.4	15.3	0.056	12.2	12.5	12.2	0.984
Polyunsaturated fatty acid (g/day)	14.7	14.5	14.7	0.967	12.4	12.6	12.0	0.480
Cholesterol (mg/day)	360	406	421	0.010	326	315	291	0.053
Keys’ lipid factor †	24.8	26.9	28.2	<0.001	27.6	27.5	26.6	0.329
Crude fiber (g/day)	4.5	4.5	4.6	0.758	4.6	4.4	4.7	0.775
Vitamin A (IU/day)	1,904	2,228	2,284	0.044	1,909	2,087	2,093	0.274
Vitamin C (mg/day)	101	107	120	0.092	121	117	120	0.937
Smoking status								
Never smoker (%)	35.1	35.4	44.0		96.6	94.5	97.4	
Past smoker (%)	10.3	11.7	12.8	0.074	0.0	1.2	0.9	0.305
Current smoker (%)	54.6	52.9	43.3		3.4	4.3	1.7	
Total cigarette-year of smoking ‡	335	396	330	0.117	13	11	06	0.826
Drinking status								
Never drinker (%)	25.3	14.9	26.4		70.1	72.9	78.6	
Occasional drinker (%)	24.2	29.9	23.6	0.645	26.4	23.9	20.1	0.054
Daily drinker (%)	50.5	55.2	50.0		3.4	3.2	1.3	
Heavy drinker § (%)	15.5	10.8	9.2	0.150	0.0	0.0	0.0	
Underweight § (%)	22.7	9.0	5.7	<0.001	19.5	10.2	7.9	0.006
Past histories of diseases								
Peptic ulcer (%)	9.3	19.7	12.8	0.687	6.9	4.3	4.8	0.588
Liver disease (%)	4.1	3.1	2.8	0.853	2.3	2.0	2.6	0.631
Diabetes (%)	3.1	4.0	5.0	0.472	2.3	2.4	2.2	0.926
Anemia (%)	3.1	2.2	1.4	0.381	18.4	13.7	10.0	0.043
Kidney disease (%)	1.0	3.6	5.0	0.110	4.6	4.3	1.3	0.062
Hypertension (%)	8.2	13.5	18.4	0.025	5.7	12.5	22.3	<0.001
Surgery (%)	7.2	7.2	5.7	0.609	4.6	7.5	4.8	0.714
Transfusion (%)	8.2	5.4	2.1	0.031	3.4	2.7	2.6	0.724
Biologic factors								
Systolic blood pressure (mmHg)	128	129	133	0.020	121	125	138	<0.001
Diastolic blood pressure (mmHg)	79.1	79.3	81.9	0.048	72.0	73.9	80.8	<0.001
LDL-Cholesterol ¶ (mg/dl)	78.7	109	142	<0.001	80.3	111	154	<0.001
HDL-Cholesterol ¶ (mg/dl)	48.2	47.9	50.2	0.419	50.7	53.1	53.9	0.186
Triglyceride (mg/dl)	95.2	116	162	<0.001	64.1	76.3	102.5	<0.001
Glucose (mg/dl)	93.0	96.5	98.8	0.008	87.6	93.4	98.0	<0.001
Total protein (g/dl)	7.2	7.2	7.4	0.005	7.3	7.4	7.5	<0.001
Hemoglobin (g/dl)	14.7	15.1	15.5	<0.001	12.2	12.7	13.2	<0.001

* ANOVA for continuous variables and chi-square test for proportional variables were conducted separately in males and females for linear trends.

† Keys’ lipid factor was calculated as (S - 0.5P) × 2.430/E+1.5(1,000 C/E)^1/2^. S, saturated fatty acid; P, polyunsaturated fatty acid; E, energy; C, cholesterol.

‡ Total cigarette-years of smoking was calculated as (P × Y); P, numbers of cigarettes smoked per day; Y, years of cigarette use.

§ Underweight was BMI <18.5 kg/m^2^; Heavy drinker was the subject with ≧56 g/day alcohol consumption.

¶ LDL-cholesterol, low density lipoprotein cholesterol; HDL-cholesterol, high density lipoprotein cholesterol.

In Table 2 (model 1), the multivariate odds ratios of having low cholesterol adjusted for age and selected lifestyle factors were 0.70 (95% confidence interval (CI): 0.52-0.94) for 1SD increment of Key’s lipid factor, 0.71 (0.51-0.97) for 1SD increment of vitamin A intake, 2.23 (1.01-4.91) for heavy drinking and 2.80 (1.21-6.46) for underweight in males, and 1.04 (1.00-1.08) for 10 cigarette-years increase in smoking in females. Even when further adjusted for BMI, these associations were still significant except for those being underweight in males. Even if we used energy-adjusted intakes of vitamin A as an independent variable instead for crude intake values of vitamin A, these results were not substantially different. When we used vitamin C as an independent variable instead of vitamin A, the intake of vitamin C was not associated with low serum TC in either gender.

**Table 2.** Multivariate odds ratios and 95% confidence intervals for having low serum total cholesterol according to lifestyle factors in males and females, Konan Town, Shiga, Japan, between 1987-1995 with the exception of 1994, Model 1.

Covariates	Low serum total cholesterol (<160 mg/dl)		

	Odds ratio † (95% CI)	Odds ratio ‡ (95% CI)	Odds ratio § (95% CI)
Males Number of subjects = 461			
Keys’lipid factor (1SD, 7.3 unit) ¶	0.73 (0.54, 0.97) *	0.70 (0.52, 0.94) *	0.71 (0.52, 0.95) *
Vitamin A intake (1SD, 1,200 IU/day)	0.71 (0.52, 0.98) *	0.71 (0.51, 0.97) *	0.72 (0.52, 0.99) *
Heavy drinking (yes vs no) #	2.15 (0.98, 4.73)	2.23 (1.01, 4.91) *	2.40 (1.07, 5.40) *
Underweight (yes vs no) #	2.50 (1.10, 5.66) *	2.80 (1.21, 6.46) *	— —
Total cigarette-years of smoking (10 cigarette-year) **	1.00 (0.99, 1.00)	1.00 (0.99, 1.01)	1.00 (0.99, 1.01)
Females Number of subjects = 571			
Keys’lipid factor (1SD, 7.6 unit) ¶	0.98 (0.74, 1.30)	0.88 (0.66, 1.18)	0.84 (0.63, 1.14)
Vitamin A intake (1SD, 1,088 IU/day)	0.84 (0.62, 1.14)	0.77 (0.56, 1.07)	0.81 (0.59, 1.13)
Heavy drinking (yes vs no) #	0.01 (0.00, 0.00)	0.01 (0.00, 0.00)	0.00 (0.00, 0.00)
Underweight (yes vs no) #	1.43 (0.59, 3.47)	1.67 (0.65, 4.30)	— —
Total cigarette-years of smoking (10 cigarette-year) **	1.02 (0.99, 1.06)	1.04 (1.00, 1.08) *	1.04 (1.00, 1.08)

* P<0.05. Multiple logistic regression analyses were performed. Yes=1, no=0 for low serum total cholesterol.

† Multivariate adjusted for selected lifestyle factors.

‡ Multivariate adjusted for selected lifestyle factors and age.

§ Multivariate adjusted for selected lifestyle factors, age and BMI except for underweight.

¶ Keys’ lipid factor was calculated as (S - 0.5P) × 2,430/E+1.5(l,000 C/E)^1/2^. S, saturated fatty acid; P, polyunsaturated fatty acid; E, energy; C, cholesterol.

# Heavy drinking was alcohol consumption ≧56 g/day; Underweight was below 18.5 kg/m^2^.

** Cigarette-year was calculated as (P × Y); P, numbers of cigarettes smoked per day; Y, years of cigarette use.

In Table 3 (model 2), the multivariate odds ratios of having low cholesterol adjusted for age and selected past history of disease were 2.59 (95% CI: 1.01-6.61) for having past history of blood transfusion and 0.42 (0.20-0.92) for having past history of peptic ulcer in males. When further adjusted for BMI, the odds ratio for blood transfusion did not reach statistical significance (P=0.067), but the odds ratio for peptic ulcer was still significant (P=0.018).

**Table 3.** Multivariate odds ratios and 95% confidence intervals for having low serum total cholesterol according to past history of disease in males and females, Konan Town, Shiga, Japan, between 1987-1995 with the exception of 1994, Model 2.

Covariates	Low serum total cholesterol (<160 mg/dl)		

	Odds ratio † (95% CI)	Odds ratio ‡ (95% CI)	Odds ratio § (95% CI)
Males Number of subjects = 461			
Peptic ulcer (yes vs no)	0.43 (0.20, 0.92) *	0.42 (0.20, 0.92) *	0.39 (0.18, 0.85)
Diabetes (yes vs no)	0.75 (0.20, 2.77)	0.74 (0.20, 2.75)	0.83 (0.22, 3.15)
Liver disease (yes vs no)	1.98 (0.57, 6.92)	1.97 (0.56, 6.90)	1.65 (0.45, 6.11)
Anemia (yes vs no)	1.75 (0.43, 7.13)	1.74 (0.43, 7.10)	1.28 (0.30, 5.44)
Kidney disease (yes vs no)	0.15 (0.02, 1.25)	0.15 (0.02, 1.24)	0.18 (0.02, 1.67)
Hypertension (yes vs no)	0.51 (0.23, 1.11)	0.49 (0.22, 1.12)	0.63 (0.27, 1.44)
Surgery (yes vs no)	0.87 (0.35, 2.15)	0.86 (0.35, 2.14)	1.11 (0.43, 2.86)
Transfusion (yes vs no)	2.60 (1.02, 6.63) *	2.59 (1.01, 6.61) *	2.32 (0.88, 6.09)
Females Number of subjects = 571			
Peptic ulcer (yes vs no)	1.47 (0.57, 3.81)	1.92 (0.73, 5.08)	1.67 (0.62, 4.51)
Diabetes (yes vs no)	1.32 (0.27, 6.34)	2.09 (0.42, 10.4)	2.14 (0.42, 10.8)
Liver disease (yes vs no)	1.04 (0.22, 4.92)	1.43 (0.29, 7.11)	1.22 (0.24, 6.15)
Anemia (yes vs no)	1.51 (0.81, 2.80)	1.44 (0.77, 2.71)	1.34 (0.71, 2.54)
Kidney disease (yes vs no)	1.88 (0.58, 6.08)	2.09 (0.62, 7.01)	1.90 (0.56, 6.44)
Hypertension (yes vs no)	0.30 (0.12, 0.76) *	0.49 (0.18, 2.32)	0.60 (0.22, 1.62)
Surgery (yes vs no)	0.89 (0.30, 2.69)	0.71 (0.23, 2.21)	0.63 (0.20, 1.99)
Transfusion (yes vs no)	0.99 (0.27, 2.63)	1.66 (0.44, 6.30)	1.40 (0.37, 5.39)

* P<0.05. Multiple logistic regression analyses were performed. Yes=1, no=0 for low serum total cholesterol, peptic ulcer, diabetes, liver disease, anemia, kidney disease, hypertension, surgery and transfusion.

† Multivariate adjusted for selected past history of disease.

‡ Multivariate adjusted for selected past history of disease and age.

§ Multivariate adjusted for selected past history of disease, age and BMI.

In Table 4 (model 3), the multivariate odds ratios of having low cholesterol adjusted for age and selected biologic factors were significantly lower in both genders. The odds ratios (95% CI) for 1SD increment of each selected biologic factor were 0.64 (0.49-0.83) for hemoglobin and 0.53 (0.33-0.82) for triglyceride in males, and 0.66 (0.52-0.83) for hemoglobin, 0.34 (0.22-0.54) for triglyceride, 0.71 (0.53-0.95) for HDL-cholesterol, 0.76 (0.59-0.97) for total protein and 0.40 (0.22-0.72) for glucose in females. Even when further adjusted for BMI, these associations were still significant.

**Table 4.** Multivariate odds ratios and 95% confidence intervals for having low serum total cholesterol according to biologic factors in males and females, Konan Town, Shiga, Japan, between 1987-1995 with the exception of 1994, Model 3.

Covariates	Low serum total cholesterol (<160 mg/dl)		

	Odds ratio † (95% CI)	Odds ratio ‡ (95% CI)	Odds ratio § (95% CI)
Males Number of subjects = 461			
HDL-C ¶ (1SD, 19.4 mg/dl)	0.85 (0.66, 1.10)	0.88 (0.68, 1.14)	0.79 (0.60, 1.04)
Hemoglobin (1SD, 1.4 g/dl)	0.71 (0.56, 0.90) *	0.64 (0.49, 0.83) *	0.68 (0.52, 0.89) *
Triglyceride (1SD, 95.4 mg/dl)	0.52 (0.33, 0.82) *	0.53 (0.33, 0.82) **	0.60 (0.38, 0.93) *
Total protein (1SD, 0.4 g/dl)	0.95 (0.74, 1.22)	0.92 (0.72, 1.19)	0.93 (0.73, 1.20)
Glucose (1SD, 16.9 mg/dl)	0.74 (0.55, 1.00) *	0.76 (0.56, 1.02)	0.81 (0.61, 1.08)
Systolic blood pressure (1SD, 17.4 mmHg)	0.92 (0.72, 1.18)	1.00 (0.77, 1.31)	1.07(0.82, 1.40)
Females Number of subjects = 571			
HDL-C ¶ (1SD, 19.0 mg/dl)	0.67 (0.50, 0.90) **	0.71 (0.53, 0.95) *	0.68 (0.50, 0.93) *
Hemoglobin (1SD, 1.4 g/dl)	0.67 (0.53, 0.84) ***	0.66 (0.52, 0.83) ***	0.67 (0.52, 0.85) **
Triglyceride (1SD, 44.9 mg/dl)	0.32 (0.20, 0.50) ***	0.34 (0.22, 0.54) ***	0.38 (0.24, 0.60) ***
Total protein (1SD, 0.4 g/dl)	0.77 (0.60, 0.99) *	0.76 (0.59, 0.97) *	0.75 (0.58, 0.98) *
Glucose (1SD, 19.7 mg/dl)	0.39 (0.21, 0.69) **	0.40 (0.22, 0.72) **	0.44 (0.24, 0.79) **
Systolic blood pressure (1SD, 20.7 mmHg)	0.77 (0.56, 1.06)	0.97 (0.67, 1.40)	0.97 (0.67, 1.41)

* P<0.05; ** P<0.01; *** P<0.001. Multiple logistic regression analyses were performed. Yes=1, no=0 for low serum total cholesterol.

† Multivariate adjusted for selected biologic factors.

‡ Multivariate adjusted for selected biologic factors and age.

§ Multivariate adjusted for selected biologic factors, age and BMI.

¶ HDL-C, high density lipoprotein cholesterol.

Since the present study was continued for eight years, we examined the difference in the mean value of each nutrient intake between the first and the latter half of the study periods, but there was no significant difference.

## DISCUSSION

### Antioxidant Vitamins

There have been few studies investigating the relation between low cholesterol and antioxidant vitamins. In the present study, we found that low serum TC was associated with low intake of vitamin A in males. Dietary beta-carotene has been identified as a possible cancer-preventing agent^20^^)^. The mean plasma alpha-carotene and beta-carotene concentrations increased in parallel with increased dietary intake of fruit and vegetables^21^^)^. Plasma beta-carotene was positively related to plasma cholesterol and beta-carotene nutrient density, negatively related to body fat and plasma triglyceride, and lower concentrations of serum cholesterol were associated with lower concentrations of serum vitamin A^22^^)^. Vitamin A may act as an intracellular antioxidant and reduce carcinogens, and low serum concentrations of retinal and beta-carotene have been associated with an excess risk of cancer^20^^,^^23^^)^. Although overall mortality from cancer was associated with low mean plasma levels of carotene adjusted for cholesterol^23^^,^^24^^)^, these studies did not include the intake of vitamin A on the basis of a nutritional survey.

On the other hand, the blood TC level may influence the availability of cholesterol to cells. Cholesterol is a component of the cell membrane and die fluidity of the cell membrane influences functions such as activity of cell membrane receptors, ion transport and immune function. Cell cholesterol is derived from endogenous synthesis and from uptake of cholesterol-rich lipoproteins from tissue fluid, and ultimately from plasma^25^^)^. Blood lipoprotein levels highly correlated with TC. Cholesterol itself, or some fat-soluble vitamins or other detoxifying agents carried in lipoproteins may play a direct role in immune response. A disease episode in a person with low TC may be more severe than the same episode in a person with high TC^6^^)^. Whether low serum TC in Japanese males promotes carcinogens or suppresses the immune system should be further investigated.

Vitamin C is also an antioxidant agent. In the present study, males with low serum TC also showed a lower mean intake of vitamin C. Since there was a significant moderate correlation between the intakes of vitamins A and C, we did not use these vitamins as independent variables at the same time. In the logistic regression model with vitamin C instead of vitamin A, the intake of vitamin C was not associated with low serum TC. However, it is worthwhile performing a further large study concerning low serum TC and vitamin C stratified by vitamin A intake levels.

### Blood Transfusion

In the present study, we found that a history of blood transfusion was associated with low serum TC when we did not adjust for BMI in males. The mean BMI of males with low serum TC was lower than those with higher serum TC (20.8 kg/m^2^ vs, 22.4 kg/m^2^, P<0.001, in t-test), This was the reason why the statistical significance disappeared in the association between low serum TC and blood transfusion when adjusted for BMI.

The prevalence of liver disease was higher in males with low serum TC although it did not reach statistical significance. The epidemiological evidence suggests that the prevalence of positive hepatitis C virus antibodies in blood donors was more than 5% in 55 - 64 of age in Osaka, which is in the same district as our study field^26^^)^. Furthermore, the infection rates of hepatitis B virus are higher in Asian populations than in Western populations^27^^)^. These former studies have suggested that the main infectious root for hepatitis was blood transfusion. The significant relation between blood transfusion and low serum TC might have possibly resulted from the latent viral hepatitis of which the Japanese have a relatively high prevalence. To our regret, since the antibodies of HBs and HCV were not checked in the present study, we could not explain the relations of these antibodies.

The diseases with liver cell destruction such as liver cirrhosis and hepatitis lead to low serum TC, whereas, fatty liver due to obesity is positively associated with high serum TC. Since it was difficult for the participants, as well as the interviewers, to classify ‘liver disease’ into high/low TC inducing forms, we did not analyze by classification of liver disease into subcategories. This might be the reason why “liver disease” was not associated with low serum TC.

### Other Factors and Females

Previous studies in Western populations found that middle-aged British males with low cholesterol showed the highest proportions of low BMI (<20 kg/m^2^), current smokers and heavy drinkers, histories of bronchitis, peptic ulcer and anemia^12^^)^. Japanese-American males with TC <180 mg/dl had the highest proportions of current smoking, heavy drinking, and certain gastrointestinal conditions^11^^)^. We found for the first time that these findings hold true to a non-Western population. In the present study, a past history of peptic ulcer in males had a significantly negative association with low serum TC in contrast to the results of British males. This might be due to their different dietary habit, genetic factors or unclear reason.

A significant association between low serum TC and total cigarette-years of smoking was found in females. This may be due to chronic obstructive pulmonary disease (COPD) caused by smoking. Basili reported that patients with COPD had lower serum levels of Apo B compared with controls^28^^)^. Apo B has a function of carrying cholesterol in the blood from the liver to tissues, and low serum levels of Apo B could indirectly result in low serum TC. Furthermore, because the proportion of smokers in females was very small, they may have other adverse health characteristics associated with low serum TC.

In the present study population, females with low serum TC were clearly younger than the other two groups with higher serum TC, which suggests that many non-menopausal females were included in low serum TC group. The effect of menstruation on the serum TC level has been confirmed; Cullinane reported that the alterations in plasma volume account for approximately half of the increase in TC during the menstrual cycle^29^^)^. This is one reason why the relation between low TC and excess mortality in females was difficult to detect in several cohort studies.

The subjects with low serum TC had lower levels of biological factors such as LDL-cholesterol, triglyceride, total protein, glucose and hemoglobin. These findings might suggest a kind of malnutrition, which was one of the common functions associated with the low serum TC.

### Study Limitations

The limitations of this study are the use of the self-reported health status concerning past history of disease and conducting the survey over eight years. Surveys using a self-reported health status might underestimate or overestimate the prevalence of a past history of disease due to the lack of an accurate diagnosis. However, in the present study, only blood transfusion had a significantly positive association with low serum TC, and overestimating the prevalence of blood transfusion is considered to be rare when using the self-reported method. The long survey period may have influenced the results of this study. For example, the on going Westernization of lifestyle in Japan may have led to a change in their dietary habit. However, the present results showed that there was no significant difference in the mean value of each nutrient intake between the first four years and the latter four years of the study period.

In conclusion, the low serum TC in the present population was significantly associated with low intake of vitamin A, heavy drinking, being underweight and having undergone blood transfusion for males. These associations may partly account for the previously reported excess numbers of deaths due to diseases from a variety of causes in Japanese males^7^^,^^30^^)^. However, these associations were not clear except for a positive association for cumulative smoking in females. It is worthwhile doing further studies on lifestyle factors and past history of disease associated with low serum TC in females.
